# BLACK-WHITE DIFFERENCES IN CHRONIC STRESS: DOES APPRAISAL MATTER FOR ANXIETY AND DEPRESSIVE SYMPTOMS?

**DOI:** 10.1093/geroni/igz038.688

**Published:** 2019-11-08

**Authors:** Lauren Brown, Leah Abrams, Uchechi Mitchell, Jennifer Ailshire

**Affiliations:** 1 University of Michigan, Ann Arbor, Michigan, United States; 2 University of Michigan School of Public Health, Ann Arbor, Michigan, United States; 3 School of Public Health, University of Illinois at Chicago, Chicago, Illinois, United States; 4 University of Southern California, Los Angeles, California, United States

## Abstract

Prior research has suggested that exposure to objectively stressful events contributes to mental health disparities in older adulthood. Yet, in order to understand the extent to which some groups bear a disproportionate stress and mental health burden, we consider black-white differences in not only stress exposure, but also stress appraisal—how upsetting the exposures are perceived to be across five domains (health, financial, residential, relationship and caregiving). Data come from 6,019 adults ages 52+ from the 2006 Health and Retirement Study. Fully adjusted models show stress exposure and appraisal significantly and independently predicted anxiety and depressive symptoms. Race and stress exposure interactions show that exposure differently predicts anxiety and depressive symptoms while race and appraisal interactions show blacks and whites report similar increases in anxiety and depressive symptoms. Findings suggest stress exposure has varying consequences for mental health of whites and blacks, while stress appraisals have similar consequences across groups.

